# Cannabidiol: A Potential New Alternative for the Treatment of Anxiety, Depression, and Psychotic Disorders

**DOI:** 10.3390/biom10111575

**Published:** 2020-11-19

**Authors:** María S. García-Gutiérrez, Francisco Navarrete, Ani Gasparyan, Amaya Austrich-Olivares, Francisco Sala, Jorge Manzanares

**Affiliations:** 1 Neurosciences Institute, University Miguel Hernández-CSIC, Avda de Ramón y Cajal s/n, San Juan de Alicante, 03550 Alicante, Spain; maria.ggutierrez@goumh.umh.es (M.S.G.-G.); fnavarrete@umh.es (F.N.); agasparyan@umh.es (A.G.); aaustrich@umh.es (A.A.-O.); fsala@umh.es (F.S.); 2 Subject Area Network of Cooperative Health Research (RETICS), Network for Addiction Disorders, Health Institute Carlos III, MICINN and FEDER, 28029 Madrid, Spain

**Keywords:** cannabidiol, depressive disorders, PTSD, anxiety disorders, schizophrenia, clinical trials, animal studies

## Abstract

The potential therapeutic use of some *Cannabis sativa* plant compounds has been attracting great interest, especially for managing neuropsychiatric disorders due to the relative lack of efficacy of the current treatments. Numerous studies have been carried out using the main phytocannabinoids, tetrahydrocannabinol (THC) and cannabidiol (CBD). CBD displays an interesting pharmacological profile without the potential for becoming a drug of abuse, unlike THC. In this review, we focused on the anxiolytic, antidepressant, and antipsychotic effects of CBD found in animal and human studies. In rodents, results suggest that the effects of CBD depend on the dose, the strain, the administration time course (acute vs. chronic), and the route of administration. In addition, certain key targets have been related with these CBD pharmacological actions, including cannabinoid receptors (CB_1_r and CB_2_r), 5-HT_1A_ receptor and neurogenesis factors. Preliminary clinical trials also support the efficacy of CBD as an anxiolytic, antipsychotic, and antidepressant, and more importantly, a positive risk-benefit profile. These promising results support the development of large-scale studies to further evaluate CBD as a potential new drug for the treatment of these psychiatric disorders.

## 1. Introduction

Mental health is currently a major public health challenge worldwide. Approximately one in four people experience some type of mental health problem at least once in their lives. Using disability adjusted life years (DALYs—years lost to ill health and premature death) as the basic measure of impact, mental health problems accounted for 19.5% of the global burden of disease [1]. Depression, alcohol use disorders, and suicide rank in the top 20 causes of DALYs lost due to all diseases at all ages [2]. In many countries, neuropsychiatric disorders account for 35% to 45% of absenteeism at work and are often associated with human rights violations, discrimination and stigma [3].

One major consequence of mental disorders is suicide. Major depressive disorder (MDD), bipolar disorder, schizophrenia (SCZ), and alcohol-use disorders (AUD) are the main risk factors for suicide [4,5,6], which takes approximately 800,000 lives each year. Suicide is the second leading cause of death in people aged 15 to 29 years, and the first in men under 40 years [7].

Limited access to mental health services and to pharmacological and psychotherapeutic treatments, especially in low- and middle-income countries, is a huge problem for these patients. Furthermore, their reluctance to seek help due to fear of being rejected by their family, friends, and community continues to be an obstacle to achieving the highest standard of mental health and well-being.

In contrast to other human diseases, neuropsychiatric disorders are not diagnosed on the basis of objective biological measures, but rather a list of symptoms, according to the Diagnostic and Statistical Manual of Mental Disorders, Fifth Edition, (DSM-V) or to the International Classification of Diseases, Tenth revision (ICD-10). These procedures result in a high degree of heterogeneity among patients diagnosed with the same psychiatric disease [8]. In part, this is because different psychiatric disorders share common symptoms and display high comorbidity, making it difficult to reach an accurate diagnosis. Together with these problems, current pharmacological and psychotherapeutic treatment options present low efficacy, particularly in medium- to high-severity cases [9,10,11]. The limited knowledge of the neurobiological mechanisms underlying neuropsychiatric diseases makes the pharmacological treatment unspecific, so the same drug groups are used for different mental disorders.

Efforts have been made to characterize the etiopathogenesis of mental disorders and to identify potential biomarkers to guide diagnosis, prognosis and the development of potential new drugs. In this respect, translational research and the advent of new technological approaches, such as neuroimaging and “omics” techniques are driving advances [12].

Thanks to these types of research, it has been possible to identify new neurotransmission systems involved in psychiatric disorders, such as the glutamatergic [13,14,15], GABAergic [16,17,18], and endocannabinoid systems (ECS) [19,20,21,22]. Some of these findings have led to the development and marketing of drugs with new mechanisms of action, such as esketamine, a non-competitive *N*-methyl-d-aspartate (NMDA) receptor antagonist, approved as therapy for treatment-resistant depression in adults in the USA and Europe [23,24,25]. Another potential drug attracting attention is cannabidiol, one of the major compounds present in the plant *Cannabis sativa* [26]. Animal models have shown that cannabidiol (CBD) displays anxiolytic, antidepressant, antipsychotic, antiepileptic and neuroprotective properties, suggesting its potential therapeutic use for several psychiatric, neurological and drug-use disorders. CBD was approved in 2018 by the U.S. Food and Drug Administration (FDA) after it was shown to be effective and safe for treating seizures associated with Lennox-Gastaut syndrome or Dravet syndrome in patients aged two years and older. This has accelerated research into its use for additional disorders. In this review, we summarize the main results provided by animal models and preliminary clinical trials conducted to date on the efficacy of CBD for treating anxiety, depressive disorders, post-traumatic stress disorder (PTSD), and SCZ. The hypothesized mechanisms of action by which CBD potentially produces its effects on these disorders are also explored. Although our results support the efficacy and safety of CBD, large-scale clinical studies are required before its final approval for human clinical use in these psychiatric disorders.

## 2. Introduction to the Phytocannabinoid Cannabidiol: Chemical Structure, Pharmacokinetics and Pharmacodynamics Profile

Over the last decades, several investigations have focused on characterizing the biological and molecular bases involved in the medical properties of the plant *Cannabis sativa*. To date, approximately 120 cannabinoids have been identified and classified into 11 groups based on their chemical structure: ∆^9^-trans-tetrahydrocannabinol (∆^9^-THC), cannabigerol, cannabicromeno, cannabidiol (CBD), cannabinodiol, cannabielsoin, cannabicyclol, cannabinol, cannabitriol, and a last group in which several cannabinoids with different chemical structure are included [27]. The main compound present in the plant is Δ^9^-THC, characterized by Gaoni and Mechoulam in 1964, responsible for the reinforcing properties of cannabis [28]. CBD is the following majority compound isolated for the first time by Adams and cols. in 1940 [29], although its chemical structure was not fully characterized until 1963 [30].

### 2.1. Overview of CBD Chemical Structure 

CBD has a chemical structure similar to Δ^9^-THC; however, both differ on the spatial conformation, fact that helps to explain the differences observed in relation to their physiopharmacological properties. Δ^9^-THC presents a planar structure that allows binding to the rCB_1_. In contrast, CBD has a slightly angular structure that produces a steric hindrance that hampers its ability to bind to this receptor [31]. As consequence, CBD displays 100 times less affinity for rCB_1_ than Δ^9^-THC [32]. This may justify the absence of reinforcing properties of CBD as opposed to Δ^9^-THC [31]. In fact, data achieve to date show that CBD did not induce euphoria or intoxication in healthy volunteers [33,34,35]. Animal studies suggested that CBD may not present reinforcing properties since it did not exhibit drug abuse potential in the conditioned place preference, spontaneous withdrawal and oral self-administration, common animal models used to evaluate the abuse potential of drugs [36,37,38].

### 2.2. Overview of CBD Pharmacological Profile

During the last years, many researchers studied the potential therapeutic utility of CBD in different diseases pointing out its possible antimicrobial, immunosuppressive, antiemetic, anti-resorptive, spasmolytic, antitumor, antifibrotic, anti-inflammatory, and anticonvulsant efficacy [39,40,41,42]. Some of these properties were further explored leading to its approval for treating spasticity in multiple sclerosis (Sativex) [43] and more recently for treating seizures associated with Lennox-Gastaut or Dravet syndromes in children [44]. Furthermore, other reports suggest that CBD may be useful for treating neurodegenerative [45,46,47,48] and psychiatric disorders [39,49,50,51]. Both animal and clinical studies pointed out that CBD presents anxiolytic, antidepressant, and antipsychotic properties, this will be further explored in detail in the following sections.

#### 2.2.1. Pharmacokinetics

As the majority of phytocannabinoids, CBD presents high liposolubility (K_o/w_: 6–7) [52]. The oral administration of CBD presents a poor bioavailability (6–19%) [53] mainly due to its extensive first-pass metabolism. To increase its oral availability, it is recommended to administer CBD together with food. Other routes of administration such as inhalation or intravenous provide better concentrations and more quickly [53,54]. Table 1 summarizes the main pharmacokinetics properties of CBD.

CBD presents a high distribution (Vd: 32 L/kg) with great accumulation in brain and adipose tissues, also due to its high liposolubility [55,56,57]. CBD is metabolized in the liver by different mechanisms (including oxidation, β-oxidation, hydroxylation, glucuronide conjugation and epoxidation) [53,56,58,59,60] and eliminated in the urine unmetabolized or as a glucuronide derivative [53,59]. There are different CBD metabolites (around 53), some of them under study to determine its potential involvement in some of its actions. 

Finally, evidences about its safety and tolerability are limited to preclinical and clinical studies. No significant side effects have been described [62,63]. Diarrhea, somnolence and decreased appetite are the most commonly side effects reported in the clinical trials performed in children with Lennox-Gastaut syndrome [44].

It is also interesting to highlight that CBD is a potent competitive inhibitor of certain cytochrome P450 isoforms (CYP2C and CYP3A) increasing the risk of drug-interactions when is given together with other drugs metabolized by these enzymes [53,59,60].

#### 2.2.2. Pharmacodynamics

CBD has the peculiarity of acting on more than 65 key targets, including the serotonin 1A receptor (5-HT_1A_), the cannabinoid-related receptors G protein-coupled receptor 55 (GPR55), transient receptor potential vanilloid 1 (TRPV1), type 1 equilibrative nucleoside transporter (ETN1), fatty acid-binding protein (FABP), nuclear factor erythroid 2-related factor 2 (NRF2), voltage-activated T-type calcium channels, adenosine and glycine receptors, mu and delta opioid receptors, and voltage-dependent anion channel 1 (VDAC1), among others [64].

The first in vitro studies revealed that CBD, at sub-micromolar concentrations, acts as an antagonist of CB_1_r and as an inverse agonist of CB_2_r [65]. However, subsequent in vivo studies showed that CBD presents low affinity for both receptors [66,67,68]. CBD seems to act more like a negative allosteric modulator of CB_1_r, modifying the power and efficiency with which endogenous cannabinoids activate the receptor [69]. In contrast, some studies indicated that CBD inhibits reuptake of anandamide (AEA) and its metabolization by the fatty acid amide hydrolase (FAAH), increasing the endogenous cannabinoid tone, a mechanism suggested by which CBD may indirectly activates CB_1_r [70].

In the case of CB_2_r, CBD acts as an inverse agonist but only at very high concentrations [71]. Moreover, CBD also acts as an antagonist of cannabinoid-related receptors as the GPR55 considered one of the main targets by which CBD exerts its properties [72,73,74].

Additional targets, including key elements of the opioidergic, dopaminergic, glutamatergic and serotonergic systems have been associated with the actions of CBD. CBD inhibits the reuptake of dopamine and glutamate in vitro [75,76]. Besides, CBD is an allosteric modulator of mu and delta opioid receptors [77,78] and a partial agonist of dopamine D_2_ receptors, reinforcing its potential as an antipsychotic [79,80].

Interestingly, additional in vitro and in vivo studies revealed that CBD induces physiological responses trough 5-HT_1A_ receptors [27,81], a serotoninergic key target involved in anxiety and depression.

## 3. Role of CBD on Anxiety and Depressive Disorders: Animal and Human Studies

### 3.1. Current Scenario

Today, over 260 million people worldwide suffer from anxiety and mood disorders, affecting an estimated 25% of the European population. Apart from its high incidence, these psychiatric disorders present high rates of prevalence, leading to a substantial reduction in the quality of life and disruptions in work/school performance, family/social life and common daily activities. In fact, anxiety and mood disorders are the main mental health causes for years lived with disability (YLD), standing at 302 YLD and 850 YLD per 100,000 inhabitants in Europe, respectively [82]. Consequently, both psychiatric disorders entail high economic costs, of around EUR 170 billion per year in Europe.

According to the DSM-V, anxiety disorders are classified into generalized anxiety disorder, panic disorder, specific or social phobias and social anxiety disorder (SAD) [83]. All types share common symptoms, including feelings of uneasiness, panic and fear; sleep problems; not being able to stay calm; being cold and/or sweaty; shortness of breath; heart palpitations; dry mouth; nausea; and avoidance of situations. Depressive disorders present high complexity and may be classified into disruptive mood dysregulation disorder (MDD), persistent depressive disorder (dysthymia), premenstrual dysphoric disorder, substance/medication-induced depressive disorder, associated with another medical condition, other specified depressive disorder and unspecified depressive disorder. Patients suffering from depressive disorders experience emotional, cognitive, physical and behavioral alterations including sadness, anxiety, guilt, irritability, impaired memory, thoughts of death and suicide, loss of motivation, disturbed sleep or appetite, tiredness, neglect of responsibilities, changes in personal appearance, and withdrawal from others [84]. The severity of depressive disorders, assessed by clinician-administered depression assessment scales, such as the Hamilton Depression Rating Scale, varies from one patient to another, with moderate-severe cases presenting worse prognosis [85]. Anxiety and depressive disorders are strongly associated with high rates of comorbidity (around 50%) [86], which can reach 90% in psychiatric patients [87,88]. Comorbidity worsens clinical management and consequently, prognosis; it increases resistance to treatment and recurrence, and it dramatically heightens the risk of suicide.

From a pharmacological point of view, anxiolytics and antidepressants are used in the clinical management of both mental disorders. For instance, benzodiazepines, the most common anxiolytic drug, is useful at the beginning of pharmacological treatment of depressive disorders [89]. Similarly, buspirone (a serotonin 5-HT_1A_ receptor agonist) is an anxiolytic for treating depressive disorders [90]. Antidepressants, especially selective serotonin reuptake inhibitors, are the most commonly used first-line treatment for anxiety disorders [91]. Although neurochemical alterations underlying anxiety and depression still remain to be elucidated, the beneficial effects found during the co-administration of both types of drugs suggest the involvement of shared neurobiological pathways.

Despite the available pharmacological treatment options, efficacy is limited, especially for preventing relapse and recurrence [92]. For example, one in three patients diagnosed with MDD develops resistance to antidepressant drugs. More importantly, current pharmacological treatments do not improve the cognitive dysfunctions associated with this mental disorder, even when combined with psychotherapy [93,94]. Conversely, the side effects of these medications, such as weight gain, loss of sexual desire and others, affect the risk-benefit ratio. Therefore, it is necessary to find new pharmacological alternatives to improve treatment outcomes for such psychiatric disorders without the burden of disabling side effects. In this respect, published animal and clinical studies, whose main results are detailed below, provide information supporting the anxiolytic and antidepressant properties of CBD.

### 3.2. Results from Animal Studies

The potential anxiolytic and antidepressant properties of CBD have been examined in several animal models since the late 1970s. Although preliminary findings were contradictory [95,96], subsequent dose-response studies showed that CBD induced an anxiolytic-like effect, which followed an inverted U-shaped curve, resulting effective at intermediate doses but not at low or high doses [97,98,99]. CBD also attenuated physiological and behavioral responses to stressful situations, reducing restraint stress along with cardiovascular and anxiogenic-like responses [100] by blocking the activation of the hypothalamus-pituitary-adrenal (HPA) axis [101] and activating the 5-HT_1A_ receptor [100]. Additional results from studies carried out using the Vogel-conflict and the marble-burying tests showed that CBD reduced anxiety- and compulsive-like behaviors, respectively [49,102,103] (Table 2). Curiously, cannabinoid CB_1_ receptor (CB_1_r), but not 5-HT_1A_ receptor, appears to mediate such effects [104]. The administration of CBD also abolished anxiety-like behavior, hyperthermia and hyperlocomotion induced by tetrahydrocannabinol (THC), modifying c-Fos expression in brain regions (medial preoptic nucleus and lateral periaqueductal gray) [105]. In contrast, CBD failed to modify anxiety induced by repeated administration of THC [106].

Complementary results indicated that the strain and pattern of administration (single and repeated) may affect CBD actions. In male C57BL/6 mice and spontaneously hypertensive rats, CBD failed to induce any effect [99,105]. Regarding the pattern of administration, chronic treatment with CBD induced an anxiolytic-like effect, whereas acute administration did not [126]. However, another study reported the opposite results [133].

Other authors have evaluated the influence of age and gender in CBD anxiolytic effects. Chronic CBD administration produced an anxiolytic-like effect when given to mice at 5 months, but not at 3 months of age [130]. However, this group of mice significantly reduced its locomotor activity. This may act as a confounding variable when interpreting the results. In contrast, in another study no effects were found in either adolescent or in adult male mice. Moreover, CBD increased anxiety in adult female mice, suggesting sex-dependent effects [128].

Administering CBD also produced interesting findings in specific brain regions. Microinjections into the bed nucleus of stria terminalis [114] and periaqueductal gray [112,113] showed anxiolytic and panicolytic-like effects, respectively. On the other hand, intracisternal or intra-prelimbic medial prefrontal cortex injection of CBD blocked the autonomic activation and anxiogenic-like responses induced by restraint stress [115,139]. These effects appear to be related with 5-HT_1A_ receptors [112,113,139].

In the fear-conditioning model, a type of associative learning task, acute administration of CBD reduced contextual fear- and anxiety-related behaviors [127,140], whereas chronic administration induced just the opposite [123]. Similarly, microinjections/infusions on specific brain regions, such as intracerebroventricular [117] and prelimbic medial prefrontal cortex, reduced freezing and anxiety [118]. However, infralimbic prefrontal cortex infusion showed contradictory results, increasing freezing [118] or facilitating fear extinction [119], depending on the total number of microinjections given. Indeed, CBD seems to disrupt aversive memory consolidation [109,120], involving anandamide, CB_1_r, CB_2_r, and peroxisome proliferator-activated receptor gamma (PPARγ) receptors in a time-dependent manner [70,119,120]. CBD also reduced the influence of PFC on corticolimbic circuits, modulating dopamine and immediate gene expression (c-fos and zif-268 proteins) [118,141], and it functionally modified the mesolimbic circuit through the direct activation of 5-HT_1A_ receptors [142,143].

The efficacy of CBD for reducing fear conditioning, together with its anxiolytic properties, stimulated the development of studies focused on evaluating its potential efficacy in animal models of PTSD, a psychiatric disorder currently classified as trauma and a stressor-related disorder. Acute or sub-chronic administration of CBD reduced the long-lasting anxiogenic-like effects induced by predator stress exposure, suggesting the intervention of 5-HT_1A_ receptors in these actions [144,145,146]. Furthermore, a new mice model of PTSD conducted by our research team showed that the administration of CBD alone or in combination with sertraline significantly reduced fear conditioning, anxiety-like behaviors and long-term gene expression alterations in the HPA axis, ECS and serotonin systems. These results support the efficacy of CBD, reducing the intense and long-lasting effects of the PTSD model [129].

Along with its anxiolytic properties, CBD displayed antidepressant efficacy in animal models of depression, inducing an antidepressant-like effect when given alone [137] or in combination with sub-effective doses of the antidepressants fluoxetine or desipramine [136], mainly through the activation of 5HT_1A_ serotonergic receptors [137]. More importantly, CBD showed a rapid and sustained antidepressant effect. A single dose of CBD induced a dose-dependent antidepressant-like effect in Swiss mice, even 7 days after its administration. Similar results were found in Flinders Sensitive and Flinders Resistant Line (FSL/FRL) rats and in Wistar rats [107]. Neuroplasticity changes were also associated, since synaptophysin, postsynaptic density protein 95 (PSD95) and brain-delivered neurotrophic factor were increased on the pre-frontal cortex (PFC) and hippocampus (HIPP) after CBD administration. This effect involves the activation of tropomyosin receptor kinase B/ mammalian target of rapamycin (TrkB/mTOR) signaling [107]. Moreover, treatment with DNA methylation inhibitors (5-AzaD and RG108) and CBD induced an antidepressant-like effect, preventing the alterations induced by stress exposure on DNA methylation and DNA methyltransferase (DNMT) activity in the HIPP and PFC [135]. This study supports the involvement of epigenetic mechanisms on CBD antidepressant properties.

Additional results indicated that the doses and the strain of rodent used may modulate the effects of CBD. In Swiss Webster mice, only the highest dose induced an antidepressant-like effect, whereas no effect was observed in DBA/2 mice [138]. Furthermore, a recent study suggested that the actions of CBD may be gender-specific, since antidepressant-like effects were found in male but not in female FSL rats [110].

Potential differences due to the pattern of CBD administration (acute vs. chronic) were also explored. In C57BL/6J mice submitted to an olfactory bulbectomy, a rodent model of depression, single and chronic administration of CBD induced anxiolytic and antidepressant-like effects. These behavioral alterations were accompanied by increases of serotonin and glutamate levels in the PFC and 5-HT_1A_ receptor function on the dorsal raphe, CA1–CA2 fields of the HIPP, amygdala and medial PFC [132]. The study further supported the involvement of the 5-HT_1A_ receptor rather than CB_1_r on CBD effects. Similar antidepressant-like effects were observed in Wistar rats [111] and Wistar–Kyoto rats as well as in a genetic model of depression [124] and in animal models displaying depressive-like symptoms such as the diabetic and normoglycemic rats [125]. Moreover, specific brain site microinjections of CBD, e.g., intra-IL or intra-prelimbic, induced antidepressant-like effects in Wistar rats involving 5-HT_1A_ and CB_1_ receptors [121].

The administration of CBD displayed antidepressant-like effects at different doses in adolescents and adult male Sprague-Dawley rats [122]. The long-lasting effects of CBD were different: 2 days for adolescent and 21 days for adult rats. Therefore, the outcome appears to depend on the age at which the CBD treatment was administered. Taken together, these findings are relevant to further explore efficacy and safety depending on the patient’s age and gender.

The effects of CBD were also evaluated for chronic unpredictable mild stress, an animal model that involved the presentation of repeated mild stressors for several weeks. Following this exposure, rodents exhibited depressive-like behavioral alterations, mainly a persistent reduction of their responsiveness to pleasurable stimuli, such as a palatable sucrose solution [147,148]. In this model, different doses and routes of CBD administration prevented anxiogenic and depressogenic-like behaviors and displayed a neuroprotective effect [116,134,149] through CB_1_r and CB_2_r [116,131]. Indeed, culture cell studies showed that CBD induced progenitor proliferation and cell cycle progression, depending on CB_1_r and CB_2_r activation and anandamide increase [149]. Overall, these results support the involvement of the endocannabinoid system in the antidepressant-like effects of CBD.

### 3.3. Results from Clinical Studies

#### 3.3.1. Clinical Studies Focused on Anxiety Disorders

The first clinical trials evaluating the anxiolytic properties of CBD were conducted in 1974 and 1982, suggesting that CBD alleviates THC-induced anxiety in healthy male volunteers [150,151] (Table 3). Subsequently, additional double-blind studies further evaluated the effects of CBD on healthy volunteers. Oral CBD administration decreased anxiety in healthy subjects exposed to the simulated public speaking test [152]. Accordingly, in another double-blind study, CBD significantly reduced subjective anxiety, evaluated by the Visual Analogue Mood Scale (VAMS), and increased mental sedation. These effects were associated with less activity on the medial temporal cluster (left amygdala-hippocampal complex, extending into the hypothalamus), and the left posterior cingulate gyrus, and with high activity on the left parahippocampal gyrus [153].

In a double-blind randomized placebo-controlled trial using a repeated-measures within-subject design in healthy volunteers who had used *Cannabis sativa* 15 times or less, CBD did not induce any behavioral or regional brain activation in the verbal learning task compared to placebo, in contrast to THC [154]. Similarly, a crossover, double-blind, repeated-measures design in 16 healthy male volunteers revealed that, unlike Δ^9^-THC, CBD did not induce psychotic symptoms, mental sedation, intellectual impairment, or physical sedation compared to placebo [35].

The anxiolytic efficacy of CBD was evaluated in patients diagnosed with anxiety disorders. In treatment-naïve patients with SAD, CBD reduced subjective anxiety, inducing changes in regional cerebral flow [155,156]. A large retrospective case series including psychiatric patients whose primary concern was anxiety or poor sleep suggested that the administration of CBD decreased anxiety rapidly and in a sustained manner. CBD also improved sleep disturbances within the first month of treatment but with fluctuations over the total three-month period evaluated [157]. However, in a clinical trial performed in non-clinical volunteers with high paranoid traits, CBD increased anxiety and had no effects on persecutory ideation in a controlled three-dimensional (3D) virtual-reality scenario [158]. These results suggest, in contrast to those observed in SAD patients, that CBD failed to display an anxiolytic-like effect in healthy volunteers with high paranoid traits.

Additional results supporting the anxiolytic properties of CBD come from clinical trials suggesting that nabiximols, a medication containing THC (2.7 mg) and CBD (2.5 mg) and used to treat spasticity in multiple sclerosis, reduced anxiety, and craving in patients with cannabis use disorder [159]. A previous case report indicated that oral CBD administration reduced cannabis withdrawal, anxiety and dissociative symptoms [166]. Besides, acute CBD vaporization improved emotional processing affect recognition and prevented the impairment of ambiguous face recognition induced by THC [160]. Similarly, in a double-blind, randomized, placebo-controlled trial in heroin users, CBD reduced anxiety and craving after its acute administration, with effects that remained stable even after 7 days [161].

Neuroimaging studies revealed that the administration of CBD altered prefrontal-subcortical connectivity during the response to fearful faces. The connection between anterior cingulate cortex-amygdala was disrupted after the administration of CBD. This finding was associated with a concurrent electrophysiological effect, pointing out both brain regions as potential key targets underlying the anxiolytic actions of CBD [162].

Additional ongoing trials have been identified. An open-label clinical trial (NCT02548559) is evaluating the effects of CBD to reduce anxiety in adults (16 participants) [167]. CBD will be given as a sublingual tincture delivered from the whole plant in a total daily dose of 30 mg for 4 weeks. Changes in anxiety behavior will be measured every week by using different scales. Following this phase 1 trial, a double-blind phase 2 clinical trial (NCT04286594) will begin following the same procedure in 75 patients diagnosed with anxiety [168].

In addition, a placebo-controlled phase 3 trial (NCT03549819) in adults is assessing the efficacy of CBD (oil capsules; flexibly dosed at 200-800 mg per day for 4 weeks) to reduce symptoms in patients diagnosed with generalized anxiety disorder, SAD, panic disorder, or agoraphobia [169]. Similarly, the goal of the pilot trial (NCT04267679) is to show the efficacy of CBD (soft gel capsules; up to a total of 100 mg/day; 12 weeks) to decrease anxiety and sleep disturbances in patients diagnosed with anxiety [170].

#### 3.3.2. Clinical Studies Focused on Stress-Related Disorders: PTSD

Today, there is a growing number of clinical trials assessing the efficacy of CBD to modulate the severity of PTSD. In an open-label clinical trial carried out in adults diagnosed with PTSD, CBD plus psychiatric medications and psychotherapy reduced the severity of PTSD symptoms after 8 consecutive weeks of treatment [163]. In addition, a double-blind randomized clinical trial (NCT04197102), designed to evaluate the efficacy of CBD (300 mg/day for 8 weeks) to reduce PTSD severity, has been recruiting patients since January 2020. Study completion is expected by May 2024 [171]. Moreover, another clinical trial, expected to finish in August 2021, is assessing the efficacy of CBD (600 mg/day for 6 weeks) for reducing alcohol intake in people with PTSD (NCT03248167) [172]. On the other hand, a placebo-controlled clinical trial (NCT02759185) is evaluating the efficacy of 4 types of smoked CBD-containing marijuana (up to 1.8 g per day for 3 weeks) for reducing symptoms severity, including anxiety and depression, in 76 military veterans with PTSD [173].

#### 3.3.3. Clinical Studies Focused on Depressive Disorders

Evidence of CBD’s antidepressant actions in humans is still scarce. In a clinical trial carried out in patients with chronic pain, high doses of nabiximols significantly reduced mood state [174]. Interestingly, oral CBD significantly decreased depressive and psychotic symptoms in cannabis users, restoring the harmful effects of cannabis on the subiculum and CA1 sub-regions of the HIPP [164]. Similar results were observed in frequent cannabis users in whom oral CBD reduced depressive- and psychotic-like symptoms and improved attentional switching, verbal learning, and memory [165]. Accordingly, nabiximols, used as an agonist replacement therapy during cannabis withdrawal, significantly reduced depression [159]. More recently, CBD users (n = 2409) reported mood-improving effects in several medical conditions in an online survey. The study did not discriminate between pure CBD and marijuana-derived CBD products with different components in the formulations [175].

An ongoing double-blind, randomized, placebo-controlled clinical trial (NCT03310593) is evaluating the effects of CBD (150–300 mg/day for 12 weeks) to reduce anxiety and depression in patients with bipolar disorder (estimated enrollment: 100 participants) [176]. The estimated study completion date is April 2022.

Taken together, these studies provide preliminary evidence supporting the efficacy and safety of CBD on these pathologies, although larger, clinical trials are needed to reach definitive conclusions.

## 4. Role of CBD on Schizophrenia

### 4.1. Current Scenario

SCZ is a heterogeneous psychiatric disorder with onset in late adolescence or early adulthood [177]. While heterogeneous, the symptoms are classified into three main categories: positive symptoms (hallucinations, delusions, disorganized thoughts, and senseless speech, bizarre behaviors); negative symptoms (social withdrawal, anhedonia, lack of emotional and facial expression, reduced speech, reduced ability to begin and sustain activities); and cognitive dysfunctions (impaired executive function, working memory and attention) [177,178]. SCZ affects only 1% of the worldwide population; however, it is a subject of intense research due to the limited efficacy of antipsychotic drugs [179]. Current treatments improve only positive symptoms following the first episode of psychosis in just 50% to 70% of patients; they present moderate efficacy for negative symptoms and have no effect on cognitive deficits [180]. At the same time, antipsychotic drugs induce severe side effects, including extrapyramidal symptoms, hyperprolactinemia, and cardiovascular complications or interval QT prolongation (depending on the type of the antipsychotic drug), limiting their chronic use [180]. New antipsychotic drugs have a better risk-benefit balance, but they still show limitations for safety and efficacy. Thus, there is a need to identify new, more effective, safer drugs for the pharmacological management of SCZ [181]. In this respect, CBD has been proposed as a new potential treatment based on findings from several preclinical studies, and more recently in clinical trials, showing its antipsychotic effects [182].

### 4.2. Results from Animal Studies

The development of animal models for complex psychiatric disorders, such as SCZ, has been instrumental in increasing our understanding of the neurobiological basis of this disorder and for identifying novel antipsychotic drugs [183]. Different experimental approaches have been used to reproduce the main features of SCZ, mostly in rodents (Table 4). Depending on the type of the manipulation used to induce these alterations, rodent models are classified into developmental models (e.g., maternal immune system’s reactivation); pharmacological models (e.g., amphetamine or ketamine administration); and lesion (e.g., neonatal ventral hippocampal lesion) or genetic (e.g., deficient functioning of the *DISC1* gene) manipulation models [183]. Jointly, they enable the reproduction of some behaviors simulating positive and negative symptoms, and to a lesser extent, cognitive impairments.

A large number of these animal models have been used to assess the potential efficacy of CBD for modulating SCZ-related behavioral and neurobiological alterations. One of the most frequent symptoms in people with SCZ is psychomotor agitation, which is pharmacologically reproduced in rodents by administering dopamine receptor agonists such as amphetamine or dexamphetamine. Antipsychotic drugs can modulate this drug-induced motor hyperactivity. In this model, a high dose of CBD reduced amphetamine- and dexamphetamine-induced motor hyperactivity, without causing additional motor effects [126,196]. Similarly, CBD normalized ketamine-motor hyperactivity when given chronically, but not acutely. Interestingly, CBD did not induce catalepsy, showing a similar profile as atypical antipsychotics [196].

Another frequent symptom in schizophrenia is the inability to filter out irrelevant stimuli or make associations for further processing, both effects that are linked to alterations in sensorimotor gating. In animal models, these alterations are measured by the pre-pulse inhibition (PPI) of the startle response, which enables the evaluation of SCZ-like behaviors and the efficacy of new potential antipsychotics. In this model, systemic or intra-nucleus accumbens (NAcc) pre-treatment with CBD attenuated the amphetamine-induced PPI alterations in Swiss mice [197]. In the same study, the authors reported similar results after pre-treatment with the anandamide hydrolysis inhibitor URB597, suggesting that the improvement achieved with CBD may be related with its ability to increase anandamide availability [67,200]. These results are consistent with those found in a clinical study, further explained in the next section of this review, studying the parallels between the improvement of SCZ-related symptoms following administration of CBD and the increase in plasma concentrations of anandamide [201]. Furthermore, similar PPI and locomotor hyperactivity normalization were found in rats pre-treated with CBD. These behavioral alterations may be associated with the regulation of mTOR/p70S6 kinase pathways phosphorylation in the NAcc shell [185].

CBD has displayed interesting effects in other SCZ animal models. Saletti et al. showed that acute CBD administration fully normalized PPI alterations induced by MK-801, a non-competitive antagonist of NMDA receptors, in capuchin monkeys (*Sapajus* spp.) [202]. Similarly, both acute [198] and chronic [192] administration of CBD modulated the PPI impairment induced by MK-801 in mice, involving, at least in part, TRPV1 receptors [67,198,203]. Chronic administration of CBD also regulated the impairments induced by MK-801 administration in social interaction and novel object recognition tests in mice, behaviors that try to simulate the negative and cognitive symptoms of SCZ. In this study, the high dose of CBD showed the same efficacy as the antipsychotic drug clozapine [193]. Moreover, social interaction and novelty object recognition tests revealed protective effects of CBD when administered after the end of the MK-801 chronic administration, in which 5-HT_1A_ receptors appear to play a relevant role [194]. Despite these promising results, in other rodent studies, CBD slightly modulated the PPI impairment induced by MK-801, without normalizing locomotor hyperactivity or social interaction [184,186]. However, pre-treatment with CBD avoided both alterations [187].

More recently, genetic animal models of SCZ, such as the mutant mice of neuregulin1 (*Nrg1 HET*), have been used to evaluate the potential antipsychotic-like effects of CBD. Neuregulin1 is a protein involved in neuronal migration, myelination and the regulation of glutamatergic NMDA and GABAergic GABA_A_ receptors expression (for a review, see [204]). Chronic administration of high doses of CBD increased social interaction in mutant mice, with a modest recovery of PPI impairment [199]. Authors identified an increase of GABA_A_ receptor binding in the granular retrosplenial cortex of the mutant mice treated with CBD, suggesting that these GABAergic receptors may be partly responsible for CBD-induced behavioral modulation. In fact, some authors have proposed that CBD may act on GABAergic and glutamatergic systems indirectly, but not exclusively, through its direct action on different targets of the ECS, serotonergic or opioid systems [205]. However, recent in vitro [206] and in vivo [207] studies suggested that CBD modulates the GABAergic system by acting directly on GABA_A_ receptors. Consequently, the effects of CBD on the GABAergic circuits may be the result of both direct and indirect modulation of this system. More studies are needed to further investigate the role of the GABAergic system in the antipsychotic-like effects of CBD.

Epidemiological human studies revealed that exposure to adverse events during pregnancy increases the risk of developing SCZ later on [208]. For this reason, the number of studies attempting to simulate SCZ-like behaviors by exposing pregnant rodents to different disturbances greatly increased in recent years. One of these models is based on the administration of polyinosinic: polycytidylic acid (poly I:C) or the anti-mitotic agent methylazoxymethanol acetate (MAM) on early gestational days (GD) to induce the activation of the maternal immune system. In mice exposed to poly I:C (GD 9 or 15), CBD normalized the increased motor activity [195] and reduced alterations on recognition, working memory, and anxiety [188]. The authors found a normalization of CB_1_r and glutamate decarboxylase in PFC and HIPP, respectively [189]. Similarly, chronic CBD administration at early ages of development modulated long-term behavioral and neurobiological consequences, including CB_1_r brain alterations, induced by MAM administration on GD 17 [191]. Previous studies suggested the involvement of CB_1_r receptors in the antipsychotic-like effects of CBD—one of the first mechanisms described [26,209]. However, the interaction between CBD and CB_1_r is controversial. On the one hand, CBD appears to activate CB_1_r by increasing anandamide levels, probably by inhibiting its reuptake and metabolism [201,210]. Conversely, some reports suggested that CBD may act as a negative allosteric modulator of CB_1_r [65,66,211,212]. Consequently, more studies are needed to further explore the role of CB_1_r on the antipsychotic actions of CBD. Similarly, the implication of CB_2_r on CBD antipsychotic-like actions could be evaluated, since this cannabinoid receptor has been related with SCZ in rodents and humans [213,214], and CBD appears to act as an inverse agonist of such receptors [65,67].

Muscarinic M1/M4 receptors and choline acetyltransferase were also associated with the modulating effects of CBD on poly I:C induced-behavioral alterations [190]. In addition, an in vitro study showed that CBD may also act on dopamine D2 receptors, inhibiting dopamine binding in the homogenized striatal tissue of rats [79]. Notably, the modulation of dopaminergic activity by CBD seems to be brain-region specific, since its administration to patients with psychosis and Parkinson’s disease modulated psychotic symptoms without worsening motor activity [215]. Thus, the selective modulation of the dopaminergic system in the striatum enable an antipsychotic effect without extrapyramidal side effects. In addition, both pre-clinical [216] and clinical [201] studies showed that, unlike typical antipsychotics, CBD does not increase plasma prolactin, adding more evidence to support its good safety profile.

Therefore, the existing scientific results suggest that CBD may be useful for modulating SCZ-related features, with a pharmacological profile similar to atypical antipsychotics [182], involving a variety of mechanisms. However, further studies are needed to increase the understanding of CBD efficacy and safety in SCZ.

### 4.3. Results from Clinical Studies

The promising results found in animal models have encouraged the development of clinical trials to evaluate its therapeutic utility for managing people who have or are at high risk of schizophrenia (Table 5). Two clinical trials evaluated the effects of chronic CBD administration [217,218] in stable antipsychotic-treated patients with SCZ. In the first, CBD did not produce changes in positive or negative symptoms, as assessed on the MATRICS Consensus Cognitive Battery (MCCB) and Positive and Negative Syndrome Scale (PANSS) in comparison with placebo. In addition, CBD failed to produce any improvement in cognitive impairments, evaluated with the MATRICS Consensus Cognitive Battery (MCCB) scale. On the other hand, CBD did not induce movement alterations, clearly a great advantage compared with current antipsychotic drugs [217]. Only sedation was significantly prevalent in the CBD-treated group compared to placebo.

In the second clinical study, a multicenter randomized controlled trial, CBD significantly improved positive psychotic symptoms (PANSS). There was also a tendency to increase cognitive performance (Brief Assessment of Cognition in Schizophrenia, BACS) and overall functioning (Global Assessment of Functioning, GAF). The administration of CBD did not modify prolactin concentrations in plasma, Simpson Angus Scale rating, weight, waist circumference, liver function, inflammatory markers, or HDL cholesterol levels—common harmful effects of current antipsychotic drugs. The prevalence of adverse events was similar in CBD- and placebo-treated patients, though there was a high proportion of mild gastrointestinal events in the CBD-treated group [218].

Similarly, in a double-blind, randomized clinical trial, CBD led to significant improvements on the PANSS scale, comparable to amisulpride, but with fewer extrapyramidal symptoms, less weight gain and a lower prolactin increase. Furthermore, CBD was well tolerated and did not significantly affect hepatic or cardiac functions. Therefore, the safety profile of CBD was better than the atypical antipsychotic amisulpride. There was also an increase in anandamide plasma concentrations in schizophrenic patients treated with CBD, highlighting this as a potential mechanism of action underlying the effects of CBD [201].

Additional clinical studies using functional magnetic resonance imaging (fMRI) showed that a single dose of CBD attenuated the reduced activity found in the mediotemporal, prefrontal and striatal brain regions of schizophrenic patients while performing verbal paired learning tasks. CBD also attenuated hippocampal-striatal functional connectivity in these patients compared to healthy controls [219]. Neuroimaging studies have also used the fMRI technique in antipsychotic-naïve patients at clinical high risk for psychosis during a verbal learning [220] or a monetary incentive delay task [221]. In the verbal learning task, a single dose of CBD improved the activation in the right caudate and in the parahippocampal gyrus and midbrain during encoding and recall, respectively [220]. In addition, CBD attenuated the hyperactivation of the left insula/parietal operculum and normalized the reaction time in the monetary incentive delay task [221]. However, CBD did not improve selective attention in schizophrenic patients, assessed by the Stroop Color Word Test. Despite these results, authors did not discard a possible beneficial effect after the chronic administration of CBD [222].

Currently, three ongoing clinical trials (NCT03883360 [223]; NCT02926859 [224]; NCT04411225 [225]) are assessing the efficacy of CBD versus placebo or olanzapine in psychosis and SCZ. The results of these clinical trials should be available in the next few years, providing evidence about the potential usefulness of CBD in psychotic disorders.

In summary, although the clinical studies are heterogeneous, the results found suggest the potential of CBD as monotherapy or as an adjunctive treatment for SCZ. However, more double-blind, placebo-controlled clinical trials are needed to evaluate effectiveness and clarify its profile of side effects.

## 5. Summary and Conclusions

Our results suggest that CBD may be a potential therapy for treating anxiety, depression, schizophrenia, and related psychotic disorders. Overall, animal models showed that the administration of CBD minimizes anxiety, depression, and stress-related behaviors. Some negative results were also found, suggesting that the anxiolytic and antidepressant properties of CBD depend on the species/strain, age, gender, doses, route of administration and time course (acute vs. chronic). Similarly, in schizophrenia and related psychotic disorders, a variety of animal models show that CBD is effective for modulating hyperactivity and PPI alterations, with a pharmacological profile similar to atypical antipsychotics [154] and the involvement of various mechanisms.

**Figure 1:**
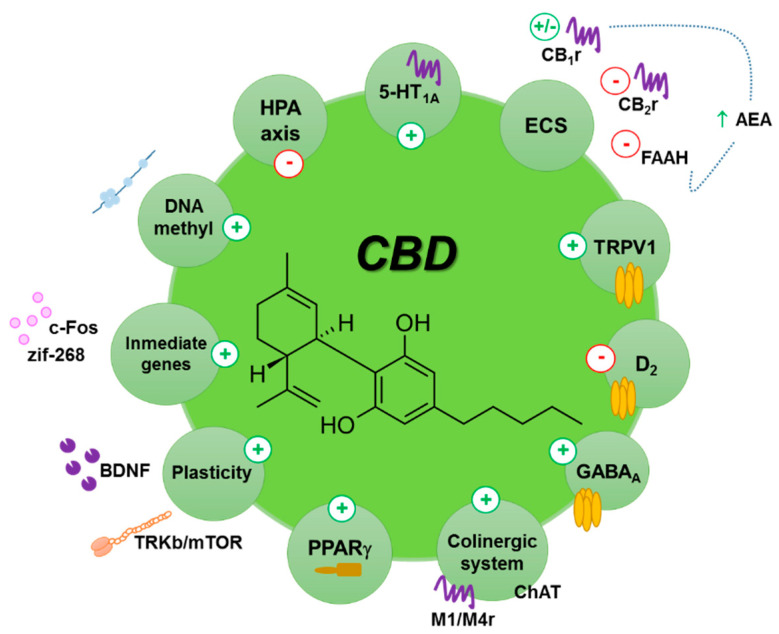
Schematic representation of the main hypothesized mechanisms described for the anxiolytic, antidepressant and antipsychotic actions of CBD. AEA: anandamide; 5-HT_1A_: serotonin receptor 1A; BDNF: brain delivered neurotrophic factor; CB_1_r: cannabinoid CB1 receptor; CB_2_r: cannabinoid CB2 receptor; ChAT: choline acetyltransferase; D2: dopamine receptor D2; DNA methyl: DNA methylation; ECS: endocannabinoid system; FAAH: fatty acid amide hydrolase; HPA axis: hypothalamus pituitary-axis; M1/M4r: muscarinic receptor 1 and 4; PPARγ: peroxisome proliferator activated receptor gamma; TRKb/mTOR: tropomyosin-receptor-kinase B/mammalian target of rapamycin; TRPV1: transient receptor potential cation channel subfamily V member 1.

One peculiarity of CBD is its multifactorial molecular profile, acting on more than 65 targets, including the 5-HT_1A_ receptor, the G protein-coupled receptor 55 (GPR55), cannabinoids receptors (CB_1_r and CB_2_r), opioid receptors (δ and μ), transient receptor potential vanilloid 1 (TRPV1), and others (for a review, see [39,64,226]). This hampers the identification of the neurobiological mechanisms by which CBD induces its behavioral effects. However, the cumulative data obtained suggest that certain targets appear to play a more relevant role than others in the anxiolytic, antidepressant and antipsychotic effects of CBD, depending on the animal model used. For example, the 5-HT_1A_ receptor plays a significant role in the anxiolytic action of CBD in some studies, but in others, using different experimental conditions, CB_1_r seems to be the most closely involved target. Despite these discrepancies, there are enough reports to conclude that both receptors, along with additional elements crucial in emotional responses and cognitive processing, such as the HPA axis, anandamide, cannabinoid CB_2_r, neurogenesis factors and GABA_A_ receptors, are involved, directly or indirectly, on the actions induced by CBD on these diseases (Figure 1). Further studies are needed to fully elucidate the mechanisms of action underlying CBD’s anxiolytic, antidepressant and antipsychotic-like effects, for example, evaluating the role of GPR55, since CBD appears to act as an antagonist of this receptor [72,73], and additional evidence supports its involvement in anxiety [227,228].

In humans, most studies have evaluated the anxiolytic-like actions of CBD in healthy volunteers or in patients with anxiety secondary to another clinical condition, such as drug use disorders. Few studies have included patients diagnosed with anxiety disorders. Besides, the small number of patients included in these studies precludes definitive conclusions. A similar scenario occurs with PTSD, where preliminary (but small) clinical trials suggest that CBD reduces PSTD severity. In the case of depressive disorders, there is a dearth of studies evaluating the effects of CBD. The efficacy of CBD for reducing depressive symptoms has only been assessed in patients with chronic pain or in cannabis users, with positive results. In the case of SCZ, a larger body of evidence suggests the possible usefulness of CBD as monotherapy or adjunctive treatment. All of the clinical trials carried out indicate that CBD is well tolerated, with no extrapyramidal side effects, less weight gain, and lower prolactin increases than current antipsychotic drugs. Thus, these results suggest that CBD presents an interesting risk-benefit profile that deserves further exploration in large clinical trials, for example, in patients of different ages, in order to ensure its safety in children and the elderly.

All of the presented results show that CBD plays a significant role in the regulation of anxiety- and depressive-related behaviors, cognition, and locomotion. However, it is necessary to develop additional, larger animal and human studies to definitively characterize the usefulness, safety, and efficacy of CBD for these psychiatric disorders. Ongoing double-blind studies, expected to finish in the next few years, will be essential to determine whether CBD is truly an option to improve the pharmacological management of these type of psychiatric patients.

## Figures and Tables

**Table 1:** Main pharmacokinetics parameters of cannabidiol (CBD).

Parameter	Values	References
K_o/w_	6–7	[52]
Oral bioavailability	6–19%	[53]
Cmax	3 ± 3.1 μg/L	[55,56,61]
Tmax	2.8 ± 1.3 h	[55,56,61]
Vd	32 L/kg	[55,56,57]
t_1/2_	1.4–10.9 h (oromucosal spray) 2–5 h (oral chronic administration) 24 h (intravenously) 31 h (smoked)	[53,54]
Plasma clearance rate	960–1500 mL/min	[53,54,55]

K_o/w_: octanol water partition coefficient; Cmax: maximum concentration; Tmax: maximum time; Vd: volume of distribution; t_1/2_: half-life.

**Table 2:** Summary of cannabidiol studies on animal models of anxiety and depression.

Strain	Doses and Route of Administration	Effect and Test	Reference
Wistar rats	1 mg/kg; i.p.; acute	Anxiolytic/SI	[99]
	2.5, 5, 10.0 mg/kg; i.p.; acute	Anxiolytic/EPM	[97]
	7–30 mg/kg; i.p.; acute	Antidepressant/FST	[107]
	5 and 15 mg/kg, i.p.; acute	No effect/SI	[99]
	1, 10, 20 mg/kg; i.p.; acute	Anxiolytic/restraint stress	[100]
	10 mg/kg; i.p.; acute 10 mg/kg; i.p.; 28 days	Anxiolytic/THC-induced conditioned emotional responses Anxiolytic/VCT Anxiolytic/CFC Antidepressant/CMS	[96,102,108]
	20 mg/kg; i.p.; acute	No effect/EPM	[97]
	3–30 mg/kg; i.p.; acute	↓ freezing behavior/CFC	[109]
	30 mg/kg; p.o.; acute	Antidepressant/FST	[110]
	30 mg/kg; i.p.; acute and chronic	Antidepressant/FST	[111]
	100 mg/kg; i.p.; acute	No effect/GSP	[95]
	30 nmol/μL; dlPAG; acute	Anxiolytic/EPM and VCT	[112]
	30 and 60 nmol/μL; PAG; acute	Anxiolytic/ETMPanicolytic/ES dPAG	[113]
	30 and 60 nmol/μL; BNST; acute	Anxiolytic/restraint stress	[114]
	30 nmol/μL; intracisternal; acute	Anxiolytic/restraint stress	[115]
	30 nmol/μL; PL; acute	Anxiolytic/EPM	[116]
	2 μg/μL; icv and mPFC; acute	↓ freezing behavior/CFC	[117]
	15 or 30 nmol/μL; IL-PFC; acute	↑ freezing behavior/CFC	[118]
	30 nmol/μL; PL-PFC; acute	↓ freezing behavior/CFC	[118]
	0.4 μg; IL-PFC; 3 days	Improve extinction/CFC	[119]
	10–30 pmol; dorsal HIP; acute	↓ Memory consolidation/CFC	[120]
	10 mg/kg; bilateral intra-PFC	↓ Memory consolidation/CFC	[109]
	10–60 nmol/side; intra-IL or intra-PL; acute	Antidepressant/FST	[121]
Sprague-Dawley rats	10 mg/kg; i.p.; 7 days 30 mg/kg; i.p.; 7 days	Antidepressant/FST	[122]
Lister-hooded rats	10 mg/kg; i.p.; 14 days	↑ Freezing behavior/CFC	[123]
Flinders Sensitive rats	7–30 mg/kg; i.p.; acute 30 mg/kg; p.o.; acute	Antidepressant/FST	[107] [110]
Flinders Resistant rats	7–30 mg/kg; i.p.; acute	Antidepressant/FST	[107]
Wistar Kyoto rats	30 mg/kg; p.o.; acute	Antidepressant/FST	[110]
	30 mg/kg; i.p.; acute	Antidepressant/SP and OR	[124]
Spontaneously Hypertensive rats	1–60 mg/kg; i.p.	No effect/SI	[99]
DBT rats	30 mg/kg; i.p.; sub-chronic	Antidepressant/FST	[125]
NGL rats	0.3 mg/kg; i.p.; acute	Antidepressant/FST	[125]
C57Bl/6J mice	1 mg/kg; i.p.; acute 1 mg/kg; i.p.; 21 days	No effect/OF and EPM Anxiolytic/LDB	[126]
	1, 10 and 10 mg/kg; i.p.; acute	No effect/CFC	[127]
	30 mg/kg; i.p.; acute	↓ freezing behavior/CFC	[127]
	5, 10 or 20 mg/kg; i.p.; acute	No effect/EPM	[128]
	10 mg/kg; i.p.; acute	No effect/OF No effect/THC-induced anxiety	[105,106]
	15 mg/kg; i.p. (+ FLX, 3 mg/kg; i.p.); acute	Anxiolytic/MBT	[103]
	20 mg/kg/day; i.p.; 3 weeks	Anxiolytic/PTSD model	[129]
	20 mg/kg; i.p.; 6 weeks 20 mg/kg; i.p.; 3 weeks	No effect/LBD and OF Anxiogenic/EPM	[130] [128]
	15, 30 and 60 mg/kg; i.p.; acute	Anxiolytic/MBT * * (even 7 days after its administration)	[104]
	30 mg/kg; i.p.; chronic 30 mg/kg; i.p.; 14 days	Anxiolytic and antidepressant/CMS Antidepressant/CMS	[131] [116]
	50 mg/kg; i.p.; 21 days	Anxiolytic/OF	[126]
	50 mg/kg; i.p.; acute	No effect/OF and EPM	[126]
	50 mg/kg; i.p.; acute 50 mg/kg; i.p.; 3 days and 10 mg/kg; 11 days	Anxiolytic/OF Antidepressant/SP	[132]
ICR mice Swiss Albino	0.5, 1, 2.5, 5, 10 and 50 mg/kg; i.p.; acute	Anxiolytic/EPM	[98]
	0.01, 0.1 and 100 mg/kg; i.p.; acute	No effect/EPM	[98]
	3 mg/kg; i.p.; acute	Anxiolytic/EPM	[133]
	10 or 30 mg/kg; i.p.; acute	No effect/EPM	[133]
	3, 10 or 30 mg/kg; i.p.; chronic	No effect/EPM	[133]
	7–30 mg/kg; i.p.; acute	Antidepressant/FST	[107]
	10 mg/kg; i.v.; 21 days 100 mg/kg; p.o.; 21 days	Antidepressant/CMS	[134]
	0.7 mg/kg; i.p.; (plus 0.1 mg/kg; i.p. 5-AZAD or RG108)	Antidepressant/FST	[135]
	7 mg/kg; i.p. (plus FLX 5 mg/kg; i.p. or DES 2.5 mg/kg; i.p.)	Antidepressant/FST	[136]
	10 mg/kg; i.p.; acute	Antidepressant/FST	[135]
	30 mg/kg; i.p.; acute	Antidepressant/FST	[137]
Swiss Webster mice	2 and 100 mg/kg; i.p.; acute	No effect/FST	[138]
	200 mg/kg; i.p.; acute	Antidepressant/FST	[138]
DBA/2 mice	2, 100 and 200 mg/kg; i.p.; acute	No effect/TST	[138]

BNST: bed nucleus of the stria terminalis; CFC: contextual fear conditioning; CMS: chronic mild stress; DBT: diabetic rats; EPM: elevated plus maze; ES dPAG: electrical stimulation dorsal periaqueductal gray; ETM: elevated T-maze; FLX: fluoxetine; FST: forced swim test; GSP: Geller-Seifter paradigm; HIP: hippocampus; icv: intracerebroventricular; IL: infralimbic; IL-PFC: infralimbic prefrontal cortex; i.p.: intraperitoneal; LDB: light dark-box; MBT: marble-burying test; mPFC: medial prefrontal cortex; NGL: normoglycemic rats; OF: open field; OR: object recognition; PAG: periaqueductal gray; PFC: prefrontal cortex; PL: prelimbic; PL-PFC: prelimbic prefrontal cortex; p.o.: oral administration; PTSD: post-traumatic stress disorder; SI: social interaction; SP: sucrose preference; TST: tail suspension test; VCT: Vogel conflict test; ↓: decrease; ↑: increase.

**Table 3:** Main outcomes achieved from clinical trials of CBD for anxiety, PTSD and depressive disorders.

Clinical Condition	Clinical Trial Design	Sample Size and Gender	Doses and Route of CBD Administration	Outcomes	References
Healthy volunteers	Double-blind randomized placebo-controlled trial	40 M (N = 5/group)	15–60 mg dissolved in ethanol and orange juice; p.o.; acute	↓ THC-induced anxiety	[150]
Healthy volunteers	Double-blind randomized placebo- and diazepam-controlled trial	8 (6 M/2 F)	0.5 mg/kg; dissolved in ethanol and artificial lemon juice; p.o.; acute	↓ THC-induced anxiety	[151]
Healthy volunteers	Double-blind randomized placebo-controlled trial	40 (18 M/22 F)	300 mg dissolved in corn oil and given in gelatin capsules; p.o.; acute	Stimulated public speaking test	[152]
Healthy volunteers	Double-blind randomized placebo-controlled trial	10 M (N = 5/group)	400 mg dissolved in corn oil and given in gelatin capsules; p.o.; acute	↓ Subjective anxiety ↑ Mental sedation	[153]
Healthy volunteers (Cannabis sativa users)	Double-blind, randomized, placebo-controlled trial, repeated-measures within-subject vs. placebo	15 M	600 mg; gelatin capsules; p.o.; 3 separate sessions	No behavioral or regional brain activation	[154]
Healthy volunteers	Double-blind, repeated-measures vs. placebo	16 M	600 mg; opaque capsules; p.o.; 3 consecutive sessions	No psychotic symptoms, mental sedation, intellectual impairment or physical sedation	[35]
Treatment-naïve SAD patients	Double-blind randomized placebo-controlled trial	10 M	400 mg dissolved in corn oil and packed inside gelatin capsules; p.o.; acute	↓ Subjective anxiety Changes in regional cerebral flow	[155]
Treatment-naïve SAD patients	Double-blind randomized placebo-controlled trial	12 M	600 mg dissolved in corn oil and packed inside gelatin capsules; p.o.; acute	↓ Subjective anxiety ↓ Cognitive impairment	[156]
Psychiatric patients with primary concern of anxiety or poor sleep	Large retrospective case series (adjunct to usual treatment)	47 anxiety (28 M/19 F) 25 poor sleep (16 M/9 F)	25 mg/day to 50–75 mg/day; capsule; 1–3 months	↓ Anxiety Improved sleep disturbances	[157]
Non-clinical volunteers with high paranoid traits	Double-blind randomized placebo-controlled trial	32 (16 M/16 F) N = 8/group	600 mg; hard gelatin capsule; p.o.; acute	↑ Anxiety No effects persecutory ideation	[158]
Cannabis use disorder	Double-blind randomized placebo-controlled trial	51 CBD N = 27 (18 M/9 F) Placebo N = 24 (21 M/3 F)	Nabiximols (CBD 2.5 mg plus THC 2.7 mg); 6 days	↓ Anxiety ↓ Craving ↓ Depression	[159]
Volunteers selected for high and low frequency of cannabis use and schizotypy (males and females	Double-blind, randomized placebo-controlled trial	48 LSS group N = 12 (9 M:3 F) LHS group N = 12 (7 M:5 F) HLS group N = 12 (11 M/1 F) HHS group N = 12 (7 M:5 F)	16 mg, formulated in alcohol solution; vaporization	Improved emotional processing	[160]
Drug-abstinent patients with heroin user disorder	Double-blind randomized placebo-controlled trial	42 CBD 400 mg N = 14 (12 M/2 F) CBD 800 mg N = 13 (11 M/2 F) Placebo N = 15 (12 M/3 F)	400 (n = 14) or 800 mg (n = 13); once daily; oral solution Epidiolex; acute (1, 2 or 24 h) and Short-term administration (3 consecutive day)	↓ Anxiety ↓ Craving ↓ Heart rate ↓ Salivary cortisol levels	[161]
Healthy volunteers	Double-blind, pseudo-randomized, placebo-controlled, repeated-measures, within-subject design	15 M	600 mg; capsules; p.o.; 3 consecutive sessions	Altered prefrontal-subcortical connectivity/response to fearful faces	[162]
PTSD	Open-label	11 (8 F/3 M)	Flexible doses: starting at 25 to 48.64 mg/day; capsule or liquid spray; 8 weeks	↓ PTSD severity	[163]
Regular cannabis users	Open-label	18 (14 M/4 F)	200 mg/day (99.5% pure crystalline of herbal origin); gelatin-coated capsules; 10 weeks	↓ Depressive ↓ Psychotic symptoms	[164]
Regular cannabis users	Open-label	20 (16 M/4 F)	200 mg/day (99.5% pure crystalline of herbal origin); gelatin-coated capsules; 10 weeks	↓ Depressive symptoms ↓ Psychotic symptoms ↑ Attentional switching ↑ Verbal learning ↑ Memory	[165]

CBD: cannabidiol; F: Female; HHS: heavy high schizotypy; HLS: heavy low schizotypy; LHS: light high schizotypy; LLS: light low schizotypy; M: Male; PTSD: post-traumatic stress disorder; SAD: social anxiety disorder; THC: tetrahydrocannabinol; ↓ reduction of; ↑ increase of.

**Table 4:** CBD studies on animal models of schizophrenia.

Strain	Doses and Route of Administration	Effect and Test	References
Wistar rats	5, 12 and 30 mg/kg; i.p.; acute	No effects on behavioral alterations induced by MK-801	[184]
Sprague Dawley rats	100 ng/0.5 µL, intra-NAcc; acute	Improve PPI and hyperlocomotion induced by AMPH	[185]
	3, 10 and 30 mg/kg, i.p.; acute	No effects on behavioral alterations induced by MK-801	[186]
	1 and 3 mg/kg; i.p.; acute	↓ anxiety and hyperlocomotion induced by MK-801	[187]
	10 mg/kg; i.p.; 11 days	Anxiolytic and ↑ recognition and working memory induced by poly I:C given on GD15	[188]
		Normalization of CB_1_r and glutamate decarboxylase alterations in the PFC and HIPP induced by poly I:C given on GD15	[189]
		Modulation of muscarinic M1/M4 receptors and choline acetyltransferase levels in PFC and HIPP/poly I:C on GD15	[190]
	10 and 30 mg/kg; i.p.; 20 days	Normalization of social withdrawal and cognitive impairment induced by MAM on GD17 Normalization of CB_1_r alterations in PFC induced by MAM given on GD17	[191]
C57BL/6J mice	1, 5, 10 and 50 mg/kg; i.p.; chronic	CBD (50 mg/kg) attenuated hyperlocomotion induced by DEXAMPH	[126]
	15, 30 and 60 mg/kg; i.p.; 21 days	Dose-dependent attenuation of MK-801-induced disruption in PPI	[192]
	30 and 60 mg/kg; i.p.; 21 days	Improvement of anxiety and cognitive impairment induced by MK-801	[193]
	15, 30 and 60 mg/kg; i.p.; 1 week	Improvement of anxiety and cognitive impairment induced by MK-801	[194]
	1 mg/kg; i.p.; 30 days	Attenuation of motor hyperactivity on PND90 induced by poly I:C given on GD9	[195]
Swiss mice	15, 30 and 60 mg/kg; i.p.; acute	CBD (30 and 60 mg/kg) blocked AMPH-induced hyperlocomotion CBD (60 mg/kg) attenuated KET-induced hyperlocomotion	[196]
	15, 30 & 60 mg/kg; i.p. 60 nmol in 0.2 µL; intra-NAcc; acute	Attenuation of PPI alterations induced by AMPH	[197]
	15 mg/kg; i.p.; acute	Modulation of PPI disruption induced by MK-801	[198]
*Nrg1 HET* mice	1, 50 and 100 mg/kg; i.p.; 21 days	CBD (50 and 100 mg/kg) improved hyperlocomotion and anxiety No significant improvement in PPI	[199]

AMPH: amphetamine; CBD: cannabidiol; DEXAMPH: dexamphetamine; GD: gestational day; HIPP: hippocampus; i.p.: intraperitoneal; KET: ketamine; MAM: methylazoxymethanol acetate; NAcc: nucleus accumbens; NAM: methylazoxymethanol acetate; *Nrg1 HET* mice: neuregulin 1 heterozygous mutant mice; PFC: prefrontal cortex; PND: postnatal day; PPI: prepulse inhibition. ↓ decrease; ↑ increase.

**Table 5:** Main outcomes achieve from clinical trials in psychosis and schizophrenia.

Clinical Condition	Clinical Trial Design	Sample Size and Gender	Doses and Route of Administration	Outcomes	Adverse Events	References
Chronic schizophrenia	Double-blind, randomized, placebo-controlled	36 CBD group N = 18 (12 M/6 F) Placebo group N = 18 (13 M:5 F)	600 mg/day; p.o.; 6 weeks	No improvement in PANSS or MCCB scores	No movement alterations	[217]
Schizophrenia or a related psychotic disorder	Double-blind randomized, placebo-controlled	88 CBD group N = 42 (28 M/14 F) Placebo group N = 44 (23 M/11 F)	1000 mg/day; oral solution; p.o.; 6 weeks	↓ Positive symptoms (PANSS) Improve cognitive performances (BACS) and overall functioning (GAF)	No prolactin or metabolic alterations; No weight gain; No liver alterations Mild GI events	[218]
Acute paranoid schizophrenia	Double-blind, randomized CBD vs. amisulpride	39 CBD group N = 20 (15 M/5 F) Amisulpride group N = 19 (17 M/2 F)	800 mg/day; p.o.; 4 weeks	↓ PANSS scores (no difference compared to amisulpride)	Fewer extrapyramidal effects Less weight gain Lower prolactin increase	[201]
Psychosis in the early stages of illness	Double-blind, randomized, placebo-controlled	34 Psychosis group N = 15 (10 M:5 F) Healthy controls N = 19 (11 M:5 F)	600 mg; gelatin capsules; p.o.; acute	Attenuation of a dysfunctional activation of mediotemporal and prefrontal cortex, and mediotemporal-striatal functional connectivity during verbal paired associate learning task	-	[219]
Patients at clinical high risk (CHR) of psychosis	Double-blind, randomized, placebo-controlled	52 Antipsychotic medication–naive participants at CHR of psychosis N = 33 (CBD group N = 16 (10 M/6 F) Placebo group N = 17 (7 M/10 F) Healthy controls N = 19 (11 M/8 F)	600 mg; gelatin capsules; p.o.; acute	Improved right caudate, parahippocampal gyrus and midbrain region’s activation during verbal learning task	-	[220]
Patients at CHR of psychosis	Double-blind, randomized, placebo-controlled	52 Antipsychotic medication–naive participants at CHR of psychosis N = 33 (CBD group N = 16 (10 M/6 F) Placebo group N = 17 (7 M/10 F) Healthy controls N = 19 (11 M/8 F)	600 mg; gelatin capsules; p.o.; acute	Attenuated the increased activation in left insula/parietal operculum, and reduced reaction time during monetary incentive delay task	-	[221]
Schizophrenia	Double-blind randomized, placebo-controlled	28 CBD 600 mg group N = 9 (5 M/4 F) CBD 300mg group N = 9 (6 M/3 F) Placebo group N = 10 (7 M/3 F)	300 or 600 mg; gelatin capsules; p.o.; acute	No effects were observed in SCWT and electrodermal responsiveness	-	[222]

BACS: Brief Assessment of Cognition in Schizophrenia; CBD: cannabidiol; GAF: Global Assessment of Functioning; CHR: clinical high risk; GI: gastrointestinal; MCCB: MATRICS Consensus Cognitive Battery; PANSS: MATRICS Consensus Cognitive Battery; p.o.: orally; SCWT: Stroop Color and Word Test. ↓ decrease; ↑ increase.
